# Whole-genome sequencing of a *Bacillus tropicus* strain carrying anthrax virulence genes isolated from a dead animal in China

**DOI:** 10.1128/mra.00505-25

**Published:** 2026-03-30

**Authors:** Bing Liang, Wei Zhou, Gejin Lu, Jie Jing, Mingwei Liu, Lin Zheng, Zixian Wang, Minghan Xu, Shiwen Sun, Lingwei Zhu, Xuejun Guo

**Affiliations:** 1 Key Laboratory of Jilin Province for Zoonosis Prevention and Control, Chinese Academy of Agricultural Sciences, Changchun Veterinary Research Institute 595703 , Changchun, China; 2 Center for Animal Disease Control and Prevention of Ordos, Ordos, China; Fluxus Inc., Sunnyvale, California, USA

**Keywords:** China, anthrax virulence genes, *Bacillus cereus *group, *Bacillus tropicus*, whole-genome sequencing

## Abstract

This report presents the complete genome sequence of an atypical *Bacillus cereus* group strain carrying anthrax virulence genes isolated in China. Based on a comparison of the reference genome sequences of each species from the *B. cereus* group, the strain was determined to be *Bacillus tropicus*.

## ANNOUNCEMENT

An atypical *Bacillus cereus* group strain, BC3, was isolated from a swab of blood from a kangaroo with anthrax-like clinical symptoms that suddenly died at Liuzhou Zoo (109°24′17.046″ E, 24°15′45.806″ N) in Guangxi Zhuang Autonomous Region, China. PCR has confirmed that this strain carries anthrax virulence genes and capsule genes but exhibits hemolytic activity and is not lysed by γ phages of *Bacillus anthracis*. The cultivation method, primer sequences, and identification method are all referenced from the WOAH Terrestrial Manual (1).

The QIAamp DNA Micro kit (Qiagen, Germany) was used to extract bacterial DNA. Qualified DNA of 0.2 μg was then sheared to 10 kb by Covaris g-TUBE (Covaris, USA) (2). Then, the SMRTbell library was constructed using the SMRTbell TM Template kit (version 1.0; PacBio, USA) (3). The SMRTbell library was purified with AMPure PB magnetic beads (PacBio), the purified sequencing library was quantified using Qubit 3.0 Fluorometer (Invitrogen, USA), and the fragment size was determined using the Agilent 2100 Bioanalyzer (Agilent Technologies). Finally, the PacBio RSII platform (PacBio) was used to obtain the whole-genome sequence (4). Subsequent genomic assembly was performed using the SMRT Portal (version 2.3.0, PacBio). The Prokaryotic Genome Annotation Pipeline algorithm (NCBI, Bethesda, MD, USA) was employed for *de novo* genome annotation (5). Genome component prediction was performed by identifying relevant coding genes for bacteria, which were obtained using the GeneMarkS program (version 4.17) (https://genemark.bme.gatech.edu/GeneMark/genemarks.cgi) (6).

**Fig 1:**
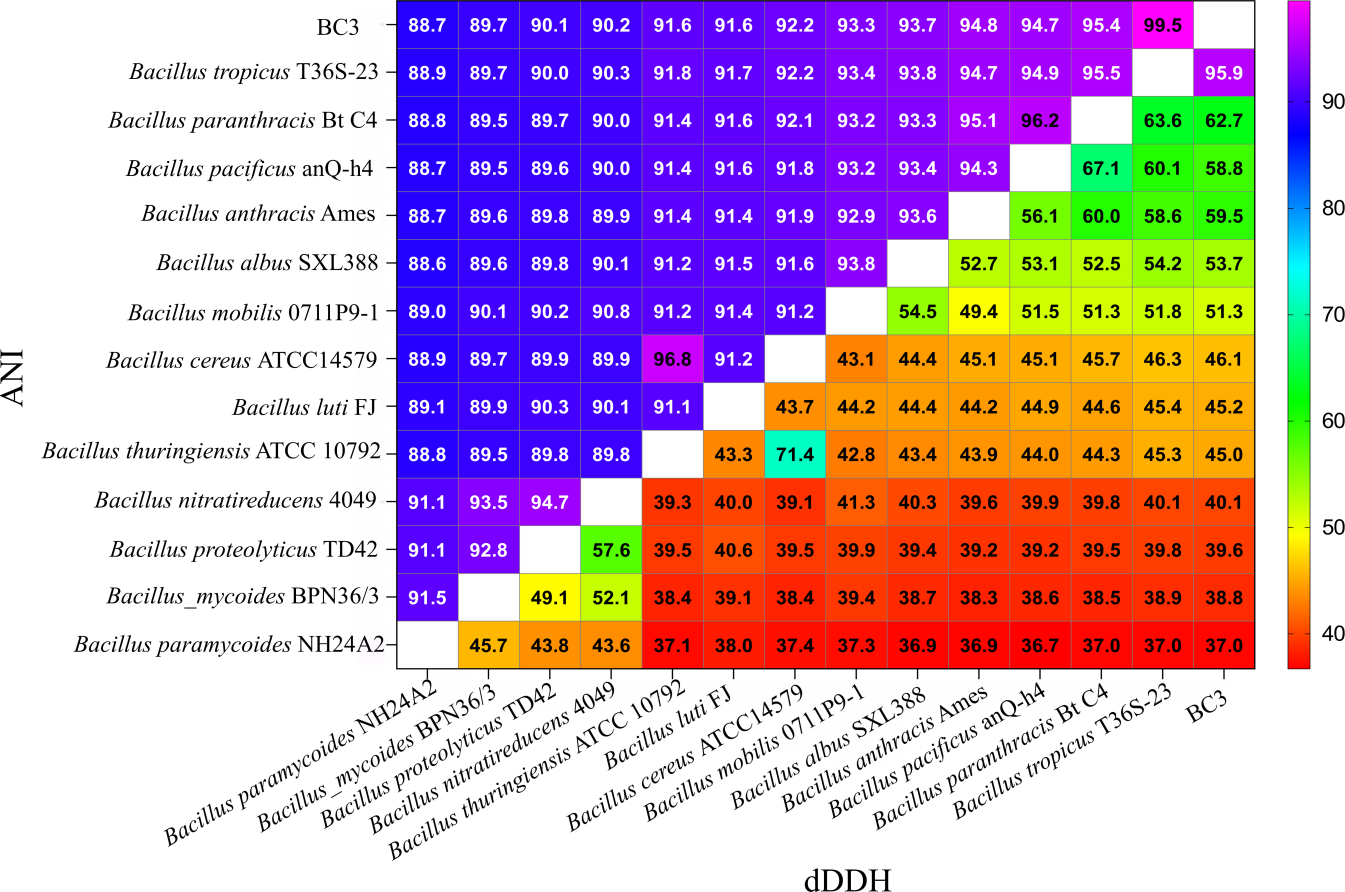
ANI and dDDH between BC3 and reference strains of various species in the *Bacillus cereus* group.

The whole-genome sequencing generated 1,299,426,954 bp of raw data. The genome size was 5,785,833 bp, with 109,392 raw reads. The scaffold N50 was 5,231,712 bp, and the final genome coverage depth reached 225×. Genome sequence analysis revealed that BC3 contains one chromosome and four plasmids, of which two plasmids, pBCXO1 and pBCXO2, exhibit high sequence homology to pXO1 and pXO2 of *B. anthracis*, respectively (Table 1). Additionally, these two plasmids harbor genes encoding the protective antigen and capsule biosynthesis genes, respectively. To determine the species of BC3, this study used the Genome-to-Genome Distance Calculator website service (http://ggdc.dsmz.de/distcalc2.php) (7) and FastANI, which is part of the GTDB Tools (https://gtdb.ecogenomic.org/tools/fastani) (8). Typically, the thresholds for classifying an isolate as the same species as the *B. cereus* group are an average nucleotide identity (ANI) value above 96% and a digital DNA-DNA hybridization (dDDH) value above 70% (9). A comprehensive whole-genome comparison using dDDH and ANI revealed a dDDH value of 95.9% and an ANI value of 99.5% between BC3 and *Bacillus tropicus* T36S-23, conclusively establishing that the BC3 strain belongs to the species *B. tropicus* (Fig. 1).

**TABLE 1:** Relevant information of BC3 whole genome

Parameter	Strain BC3	Chromosome	Plasmid			
			pBCXO1	pBC244	pBC52	pBCXO2
Accession no.	GCF_002117465.1	CP020937	CP020940	CP020938	CP020939	CP020941
Size (bp)	5,785,833	5,231,712	168,378	244,929	52,693	88,621
GC %	35.5	35.5	32.5	36.5	36.5	33
No. of genes	6,227	5,629	163	273	71	91
tRNA	119	102		17		
rRNA	37	37				
ncRNA	5	5				

## Data Availability

Raw sequences were deposited in the NCBI SRA database under SRA accession number SRR34390074, and the complete BC3 genome sequences were deposited in NCBI GenBank under the accession numbers listed in Table 1. Additionally, for confirmation of the BC3 strain, the reference strain of *Bacillus tropicus* is T36S-23, which has the accession number GCF_031357045.1.

## References

[1] World Organization for Animal Health (WOAH). 2023. Terrestrial animal health code. https://www.woah.org/fileadmin/Home/fr/Health_standards/tahm/3.01.01_ANTHRAX.pdf.

[2] Tang K, Yang Y, Lin D, Li S, Zhou W, Han Y, Liu K, Jiao N. 2016. Genomic, physiologic, and proteomic insights into metabolic versatility in Roseobacter clade bacteria isolated from deep-sea water. Sci Rep 6:35528. doi:10.1038/srep35528 27762339 PMC5071866

[3] Zhang HL, Ntambo MS, Rott PC, Chen G, Chen LL, Huang MT, Gao SJ. 2020. Complete genome sequence reveals evolutionary and comparative genomic features of Xanthomonas albilineans causing sugarcane leaf scald. Microorganisms 8:182. doi:10.3390/microorganisms8020182 32012870 PMC7074728

[4] Lü Y, Zhao S, Liang H, Zhang W, Liu J, Hu H. 2019. The first report of a novel IncHI1B bla (SIM-1)-carrying megaplasmid pSIM-1-BJ01 from a clinical Klebsiella pneumoniae isolate. Infect Drug Resist 12:2103–2112. doi:10.2147/IDR.S212333 31413597 PMC6657655

[5] Toropov V, Demyanova E, Shalaeva O, Sitkin S, Vakhitov T. 2020. Whole-genome sequencing of Lactobacillus helveticus D75 and D76 confirms safety and probiotic potential. Microorganisms 8:329. doi:10.3390/microorganisms8030329 32111071 PMC7142726

[6] Zhang W, Zhao Y, Yang G, Peng J, Chen S, Xu Z. 2019. Determination of the evolutionary pressure on Camellia oleifera on Hainan Island using the complete chloroplast genome sequence. PeerJ 7:e7210. doi:10.7717/peerj.7210 31289703 PMC6599451

[7] Kaunietis A, Buivydas A, Čitavičius DJ, Kuipers OP. 2019. Heterologous biosynthesis and characterization of a glycocin from a thermophilic bacterium. Nat Commun 10:1115. doi:10.1038/s41467-019-09065-5 30846700 PMC6405829

[8] Gómez-Sanz E, Haro-Moreno JM, Jensen SO, Roda-García JJ, López-Pérez M. 2021. The resistome and mobilome of multidrug-resistant Staphylococcus sciuri C2865 unveil a transferable trimethoprim resistance gene, designated dfrE, spread unnoticed. mSystems 6:e0051121. doi:10.1128/mSystems.00511-21 34374564 PMC8407400

[9] Liu Y, Du J, Lai Q, Zeng R, Ye D, Xu J, Shao Z. 2017. Proposal of nine novel species of the Bacillus cereus group. Int J Syst Evol Microbiol 67:2499–2508. doi:10.1099/ijsem.0.001821 28792367

